# Correlation Analysis Between Prognostic Nutritional Index Trajectory Categories and Radiotherapy-induced Severe Oral Mucositis in Head and Neck Cancer Patients Undergoing Radiotherapy

**DOI:** 10.3290/j.ohpd.b5877400

**Published:** 2024-12-12

**Authors:** Meizi Liu, Fei Gao, Ran An, Zitong Wu, Wenfeng Chen

**Affiliations:** a Meizi Liu Master Student in Nursing Science, Teaching and Research Section of Clinical Nursing, Xiangya Hospital Central South University, Changsha, Hunan Province, China; Xiang Ya Nursing School, Central South University, Changsha, Hunan Province, China. Conceptualization, Methodology, Formal Analysis, Investigation, wrote original draft, read and approved the final manuscript.; b Fei Gao Master Student in Nursing Science, Teaching and Research Section of Clinical Nursing, Xiangya Hospital Central South University, Changsha, Hunan Province, China; Xiang Ya Nursing School, Central South University, Changsha, Hunan Province, China. Investigation, reviewed, edited, read and approved the final manuscript.; c Ran An Master Student in Nursing Science, Teaching and Research Section of Clinical Nursing, Xiangya Hospital Central South University, Changsha, Hunan Province, China. Read and approved the final manuscript.; d Zitong Wu Master Student in Nursing Science, Teaching and Research Section of Clinical Nursing, Xiangya Hospital Central South University, Changsha, Hunan Province, China; Xiang Ya Nursing School, Central South University, Changsha, Hunan Province, China. Investigation, read and approved the final manuscript.; e Wenfeng Chen Associate Professor, Teaching and Research Section of Clinical Nursing, Xiangya Hospital Central South University, Changsha, Hunan Province, China. Conceptualization, reviewed and edited the manuscript, Supervision, Funding acquisition, read and approved the final manuscript.

**Keywords:** head and neck cancer, oral mucositis, prognostic nutritional index, radiotherapy trajectory categories

## Abstract

**Purpose:**

To establish the potential prognostic nutritional index (PNI) trajectory categories and examine the association between these PNI trajectory categories and severe radiotherapy-induced oral mucositis (RIOM).

**Materials and Methods:**

This was a longitudinal retrospective observational study on 470 head and neck cancer inpatients who were to undergo radiotherapy from a grade-A tertiary hospital in Hunan Province between July 2022 and October 2023. The latent class growth model and growth mixed model were used to identify PNI trajectory categories. Logistic regression analysis was used to examine the correlation between PNI trajectory categories and severe RIOM.

**Results:**

Three PNI trajectory categories were identified: “PNI high-level group” (20.9%), “PNI medium-level group” (61.3%) and “PNI low-level group” (17.9%). The risk of severe RIOM was statistically significantly higher in the “PNI medium-level group” and “PNI low-level group” with OR 2.174 (95%CI 0.980-4.822) and OR 3.45 (95%CI 1.212-9.815), respectively. PNI trajectory categories and cancer type were independent risk factors for severe RIOM.

**Conclusion:**

PNI can be used as a biomarker to predict severe RIOM. Patients with head and neck cancer who have a lower PNI are at higher risk of severe RIOM.

Cancer stands as the leading cause of death worldwide, with nearly 10 million deaths attributed to this disease in 2020.^4^ Cancers of the head and neck region are predominantly squamous cell carcinomas with over 800,000 new cases and over 400,000 deaths in 2020.^16,28^ Head and neck cancer (HNC) is an epithelial malignancy arising from the paranasal sinuses, nasal cavity, oral cavity, pharynx, larynx, and salivary glands.^14^ Radiotherapy is the mainstay of treatment for HNC, but while damaging cellular DNA to kill tumor cells, radiation can also cause radiotherapy-induced oral mucositis (RIOM).^19^ Severe RIOM presents with large areas of oral fibrous mucositis, mucosal ulceration, bleeding, and severe pain.^1,33^ In the acute phase of RIOM, patients experience pain, anorexia, dehydration, and dysphagia, leading to a decline in nutritional status and quality of life, radiotherapy dose reduction or discontinuation, prolonged hospitalisation, and increased treatment costs.^17^ It has been noted that more than half of HNC patients undergoing radiotherapy who develop RIOM are at suspected or mild levels of malnutrition, and more than one-third are severely malnourished.^32^ The principles of dental evaluation and management of HNC suggest that all patients should undergo nutritional risk assessment and receive nutritional counseling or nutritional intervention.

Molecular biomarkers derived from genetics, proteins, metabolites, autoantibodies and microbiome play key roles in clinical oncology.^15,28^ The prognostic nutrition index (PNI) was first proposed by Buzby et al^2^ in 1980 to assess the preoperative nutritional and inflammatory status of patients undergoing gastrointestinal surgery. The PNI screens individuals for nutritional risk through serum albumin levels and total lymphocyte counts in the peripheral blood. The calculation is simple, practical, and has a wide range of clinical applications, such as predicting the prognosis of cardiovascular disease^5,9^ and the severity of coronavirus disease 2019.^10^ In recent years, many researchers have confirmed the prognostic role of PNI in various cancers, e.g., gastric cancer,^12^ renal cell carcinoma,^29^ oral cancer, etc.^8^ However, few studies have reported the correlation between PNI and severe RIOM in HNC patients undergoing radiotherapy. In addition, most studies investigating the associations of PNI with disease measured the PNI only once during treatment. These study designs ignored the effects of the PNI changes over time (i.e., dynamic changes in size). The PNI of HNC patients will show different regularity characteristics and individual differences with the duration of radiotherapy. Traditional research analysis methods assume that the study population is homogeneous, and ignores group heterogeneity. The latent class growth model (LCGM) and growth mixed model (GMM) deal with group heterogeneity by dividing the heterogeneous study population into subgroups and describing the developmental trajectories of each subgroup.^20^ In this study, we used LCGM and GMM to establish the potential PNI trajectory categories and examine the association between these PNI trajectory categories and severe RIOM to provide a reference basis for preventing and treating RIOM in the early clinical stage.

## Materials and Methods

### Study Design, Participants and Setting

This was a longitudinal retrospective observational study utilising convenience sampling, which involved HNC inpatients who were to undergo radiotherapy from a grade-A tertiary hospital in Hunan Province between July 2022 and October 2023. The inclusion criteria were as follows: (1) diagnosed as HNC through pathological evaluation; (2) age ≥18 years; (3) agreeing to radiotherapy and signing the informed consent; (4) no mental or intellectual impairment. Patients were excluded if they had other malignant tumors, incomplete clinical data, or oral mucositis that already existed before radiotherapy.

Patient screening and data collection were performed by three researchers (ML, FG, and RA). The researchers collected serum albumin and total lymphocyte counts in the peripheral blood from HNC patients undergoing radiotherapy at seven time points, including baseline (before radiotherapy) and 1, 2, 3, 4, 5, and 6 weeks after beginning radiotherapy (T0, T1, T2, T3, T4, T5, and T6, respectively). The information on variables was collected from the medical information system.

### Sample Size

The sample size was calculated by referring to the table provided by Barcikovski and Robey^3^ in the single group repeated measures analysis. This study required seven repeated measures: if the mean correlation coefficient was determined to be 0.50, the effect size was 0.14, the significance level was 0.05, and finally, the sample size was 106 cases. Considering 10%-20% of missed visits and invalid questionnaires, a sample size of at least 118~133 cases was needed for this study. To improve the reliability, enhance the internal and external validity of the study, and reduce the bias due to individual differences, random errors, and other factors, the actual sample size collected in this study was larger than that based on the calculated sample size.

### Ethical Considerations

The study protocol was approved by the Xiangya Hospital Central South University, Clinical Medical Ethics Committee (protocol number 2022060874). All methods were carried out following the Declaration of Helsinki. Assurance was given that patient information would not be disclosed. Relevant information was used for this study only.

### Instruments

#### General demographics and disease-related characteristics

The general demographic data and disease-related characteristics of the participants included gender, age, body mass index (BMI), educational level, marital status, occupational status, years of smoking, years of drinking, years of betel nut chewing, place of residence, cancer type, disease duration (years), degree of differentiation, cancer stage, number of comorbidities, diabetes, surgery, simultaneous chemoradiotherapy, nasogastric intubation and radiotherapy dose.

#### Laboratory tests

The laboratory test results included leukocytes, erythrocytes, hemoglobin, platelets, neutrophils, lymphocytes, monocytes, neutrophil percentage, total protein, albumin, globulin, and creatinine.

### Outcome Definition

#### The severity of RIOM

The severity of oral mucositis was assessed according to the World Health Organization Oral Toxicity Scale (WHO Grading Scale): grade 0, no change; grade I, soreness/erythema; grade II. erythema, ulcers, can eat solids; grade III, ulcers with liquid diet only; grade IV, alimentation not possible. Among them, grade III and IV are severe oral mucositis.

#### PNI

PNI=10 Alb. + 0.005 Lymph. C., where Alb. is serum albumin level (g/100 ml) and Lymph. C. is total lymphocytes count/mm^3^ peripheral blood.^24^ The higher the PNI, the better the nutritional status, usually with 45 as the threshold. PNI ≥ 45 is considered normal or mildly abnormal nutritional status, and PNI < 45 is considered moderate or severe malnutrition.

### Data Analysis

The statistical analyses were conducted using SPSS version 25.0 and Mplus version 8.3. The continuous variables are described using means, standard deviations, medians and interquartile ranges. The categorical variables were expressed as frequencies and percentages. LCGM and GMM, which can identify distinct subgroups among heterogeneous populations, was performed to fit the PNI trajectories. The fitness of PNI trajectories was assessed by the following indicators: Akaike information criterion (AIC), Bayesian information criterion (BIC), sample size-adjust Bayesian information criterion (aBIC), entropy, Lo-Mendell-Rubin (LMR) and Bootstrapped likelihood ratio test (BLRT). Categorical variables were analyzed using a χ^2^ test while continuous variables were analysed using an independent t-test or the Mann-Whitney U-test among severe RIOM and non-severe RIOM. Logistic regression analysis was used to explore the correlation between PNI trajectory categories and severe RIOM. Statistical significance was defined as p < 0.05.

## Results

All data analysed in the study are available from the corresponding author upon reasonable request.

### Participant Characteristics and Disease-related Characteristics

A total of 470 patients with HNC who underwent radiotherapy were included in this study. Most of the patients were aged from 41 to 65 years. About half of the patients had a BMI between 18.5 and 23.9 or 24.0 and 27.9 and had full-time jobs. Most patients were male (81.3%) and married (94.7%). Of these patients, 56.6% were smokers, 33.4% drank alcohol, 17.8% chewed betel nuts. More than half of the patients were diagnosed with nasopharyngeal cancer, and 91.5% were in advanced stages (stages III and IV). 38.3% of the patients underwent surgery, and 63.0% received simultaneous chemoradiotherapy. Other information is shown in Table 2.

**Table 2:** Baseline characteristics by PNI trajectories

Variables	All (n=470)	C1 (n=98)	C2 (n=288)	C3 (n=84)	P
Gender					0.095
Male	382 (81.3%)	87 (88.8%)	227 (78.8%)	68 (81.0%)	
Female	88 (18.7%)	11 (11.2%)	61 (22.1%)	16 (19.0%)	
Age in years					
≤40	89 (18.9%)	31 (31.6%)	53 (18.4%)	5 (6.0%)	0.000
41~65	330 (70.2%)	62 (63.3%)	208 (72.2%)	60 (71.4%)	
≥66	51 (10.9%)	5 (5.1%)	27 (9.4%)	19 (22.6%)	
BMI					0.000
<18.5	25 (5.3%)	2 (2.0%)	15 (5.2%)	8 (9.5%)	
18.5~23.9	264 (56.2%)	42 (42.9%)	166 (57.6%)	56 (66.7%)	
24.0~27.9	136 (44.9%)	44 (25.7%)	74 (21.4%)	18 (28.9%)	
≥28.0	45 (9.6%)	10 (2.1%)	33 (7.0%)	2 (0.4%)	
Education level					0.000
Primary school and below	84 (17.9%)	8 (8.2%)	52 (18.1%)	24 (28.6%)	
Junior high school	156 (33.2%)	23 (23.5%)	105 (36.5%)	28 (33.3%)	
High school or junior college	142 (30.2%)	36 (36.7%)	84 (29.2%)	22 (26.2%)	
College and above	88 (18.7%)	31 (31.6%)	47 (16.3%)	10 (11.9%)	
Marital status					0.449
Married	445 (94.7%)	91 (92.9%)	274 (95.1%)	80 (95.2%)	
Unmarried	21 (4.5%)	5 (5.1%)	13 (4.5%)	3 (3.6%)	
Divorced or widowed	4 (0.9%)	2 (2.0%)	1 (0.3%)	1 (1.2%)	
Occupational status					0.001
Full-time job	217 (46.2%)	62 (63.3%)	132 (45.8%)	23 (27.4%)	
Unemployed	31 (6.6%)	5 (5.1%)	20 (6.9%)	6 (7.1%)	
Retirement	51 (10.9%)	6 (6.1%)	29 (10.1%)	16 (19.0%)	
Farmer	137 (29.1%)	21 (21.4%)	87 (30.2%)	29 (34.5%)	
Other	34 (7.2%)	4 (4.1%)	20 (6.9%)	10 (11.9%)	
Years of smoking					0.001
0	204 (43.4%)	45 (45.9%)	129 (44.8%)	30 (35.7%)	
1~10	58 (12.3%)	20 (20.4%)	35 (12.2%)	3 (3.6%)	
≥11	208 (44.3%)	33 (33.7%)	124 (43.1%)	51 (60.7%)	
Years of drinking					0.610
0	313 (66.6%)	67 (68.4%)	189 (65.6%)	57 (67.9%)	
1~10	54 (11.5%)	14 (14.3%)	33 (11.5%)	7 (8.3%)	
≥11	103 (21.9%)	17 (17.3%)	66 (22.9%)	20 (23.8%)	
Years of betel nut chewing					0.916
0	386 (82.1%)	79 (80.6%)	236 (81.9%)	71 (84.5%)	
1~10	58 (12.3%)	13 (13.3%)	37 (12.8%)	8 (9.5%)	
≥11	26 (5.5%)	6 (6.1%)	15 (5.2%)	5 (6.0%)	
Place of residence					0.077
City	201 (42.8%)	47 (48.0%)	127 (44.1%)	27 (32.1%)	
Rural	269 (57.2%)	51 (52.0%)	161 (55.9%)	57 (67.9%)	
Cancer type					0.645
Oral cancer	121 (25.7%)	25 (25.5%)	72 (25.0%)	24 (28.6%)	
Nasopharyngeal cancer	295 (62.8%)	64 (65.3%)	185 (64.2%)	46 (54.8%)	
Laryngeal cancer	21 (4.5%)	3 (3.1%)	12 (4.2%)	6 (7.1%)	
Other	33 (7.0%)	6 (6.1%)	19 (6.6%)	8 (9.5%)	
Disease duration (years)					0.381
<1	437 (93.0%)	90 (91.8%)	272 (94.4%)	75 (89.3%)	
1~3	23 (4.9%)	5 (5.1%)	11 (3.8%)	7 (8.3%)	
≥4	10 (2.1%)	3 (3.1%)	5 (1.7%)	2 (2.4%)	
Degree of differentiation					0.552
Well differentiated	69 (14.7%)	12 (12.2%)	39 (13.5%)	18 (21.4%)	
Moderately differentiated	80 (17.0%)	18 (18.4%)	46 (16.0%)	16 (19.0%)	
Poorly differentiated	91 (19.4%)	20 (20.4%)	57 (19.8%)	14 (16.7%)	
Undifferentiated	230 (48.9%)	48 (49.0%)	146 (50.7%)	36 (42.9%)	
Cancer stage					0.175
I	6 (1.3%)	3 (3.1%)	3 (1.0%)	0 (0%)	
II	34 (7.2%)	10 (10.2%)	17 (5.9%)	7 (8.3%)	
III	193 (41.1%)	45 (45.9%)	117 (40.6%)	31 (36.9%)	
IV	237 (50.4%)	40 (40.8%)	151 (52.4%)	46 (54.8%)	
Number of comorbidities					0.074
0	61 (13.0%)	12 (12.2%)	42 (14.6%)	7 (8.3%)	
1~4	268 (57.0%)	63 (64.3%)	163 (56.6%)	42 (50.0%)	
5~10	130 (27.7%)	23 (23.5%)	76 (26.4%)	31 (36.9%)	
≥11	11 (2.3%)	0 (0%)	7 (2.4%)	4 (4.8%)	
Diabetes					0.817
Yes	66 (14.0%)	15 (15.3%)	38 (13.2%)	13 (15.5%)	
No	404 (86.0%)	83 (84.7%)	250 (86.8%)	71 (84.5%)	
Surgery					0.885
Yes	180 (38.3%)	36 (36.7%)	110 (38.2%)	34 (40.5%)	
No	290 (61.7%)	62 (63.3%)	178 (61.8%)	50 (59.5%)	
Simultaneous chemoradiotherapy					0.990
Yes	296 (63.0%)	61 (62.2%)	182 (63.2%)	53 (63.1%)	
No	174 (37.0%)	37 (36.9%)	106 (36.8%)	31 (37.8%)	
Nasogastric intubation					0.002
Yes	38 (8.1%)	1 (1.0%)	24 (8.3%)	13 (15.5%)	
No	432 (91.9%)	97 (99.0%)	264 (91.7%)	71 (84.5%)	
Radiotherapy dose	69.96 (66.00, 69.96)	69.96 (66.00, 69.96)	69.96 (66.00, 69.96)	69.96 (64.50, 69.96)	0.577
Laboratory test results					
Leukocytes	5.10 (4.10, 6.40)	5.30 (4.48, 6.80)	5.09 (4.00, 6.30)	5.00 (3.90, 6.50)	0.194
Erythrocytes	4.13 (3.75, 4.58)	4.53 (4.09, 4.92)	4.11 (3.77, 4.54)	3.83 (3.45, 4.26)	0.000
Hemoglobin	125.48±16.79	134.98±16.71	124.83±15.42	116.63±16.03	0.000
Platelets	240.00 (188.00, 296.25)	235.00 (188.75, 310.00)	242.50 (193.00, 299.75)	216.50 (175.25, 283.25)	0.205
Neutrophils	3.00 (2.30, 4.00)	3.10 (2.38, 4.13)	3.00 (2.20, 3.80)	3.10 (2.30, 4.48)	0.600
Lymphocytes	1.40 (1.10, 1.70)	1.60 (1.30, 2.10)	1.40 (1.10, 1.70)	1.10 (0.90, 1.50)	0.000
Monocytes	0.50 (0.40, 0.60)	0.47 (0.40, 0.60)	0.50 (0.40, 0.60)	0.50 (0.30, 0.70)	0.839
Neutrophil percentage	59.56±10.82	57.44±10.08	59.22±10.75	63.20±11.13	0.001
Total protein	72.00 (68.55, 75.23)	74.10 (70.75, 76.60)	71.85 (68.60, 74.80)	69.90 (66.08, 73.88)	0.000
Albumin	43.30 (41.20, 45.40)	45.85 (44.00, 47.20)	43.20 (41.30, 45.00)	40.70 (37.70, 42.70)	0.000
Globulin	28.55 (25.90, 31.40)	28.05 (25.40, 31.20)	28.50 (25.90, 31.35)	29.30 (26.25, 32.30)	0.235
Creatinine	72.00 (62.30, 82.00)	72.50 (61.30, 82.50)	71.55 (62.00, 81.34)	72.70 (63.10, 90.75)	0.251


### PNI Trajectory Categories in HNC Radiotherapy Patients

The PNI of HNC patients undergoing radiotherapy at seven time points was used as an observational index. The results of the LCGM and GMM model fitting for the PNI are shown in Table 1. Firstly, LCGM was used to extract one-class to five-class sequentially (one-class: the heterogeneous populations are classified into 1 category; two-class: the heterogeneous populations are classified into 2 categories, and so on). When the number of extracted latent categories was increased from one-class to two-class, the values of AIC, BIC, and aBIC in the fitted indicators decreased, and both LMR and BLRT reached statistically significant levels (p<0.05). When increasing from two-class to three-class, the values of AIC, BIC, and aBIC in the fitted indicators decreased accordingly, and the p-value of BLRT still supporting the increase of the number of categories, the p-value of LMR is not statistically significant, but the entropy increases; when increasing from four-class to five-class, the percentage of the smallest class is less than 10%. To judge the optimal model, one-class to five-class are further extracted using GMM. In GMM, when the number of extracted latent categories increases from two-class to five-class, the percentage of the smallest class is lower than 5%. Combining the above information, three classes in LCGM are finally retained.

**Table 1:** Model fit results for PNI trajectory in HNC radiotherapy patients (n=470)

Model	AIC	BIC	aBIC	Entropy	LMR	BLRT	Proportions per class(%)
LCGM	1C	18147.984	18189.511	18157.773	-	-	-	1
2C	17166.204	17224.342	17179.909	0.827	0.0005	0.0000	0.494/0.506
3C	16742.290	16817.040	16759.911	0.870	0.0746	0.0000	0.179/0.209/0.613
4C	16554.793	16646.153	16576.329	0.841	0.4063	0.0000	0.089/0.128/0.385/0.398
5C	16457.961	16565.932	16483.413	0.856	0.2071	0.0000	0.015/0.085/0.155/0.338/0.406
GMM	1C	16297.366	16363.810	16313.029	–	–	–	1
2C	16254.673	16350.185	16277.188	0.905	0.0364	0.0000	0.034/0.966
3C	16224.650	16349.232	16254.017	0.938	0.1932	0.1053	0.009/0.036/0.955
4C	16221.579	16362.772	16254.862	0.899	0.2517	0.3333	0.002/0.023/0.055/0.919
5C	16203.778	16361.582	16240.977	0.668	0.0604	0.0882	0.002/0.066/0.143/0.174/0.615
AIC: Akaike Information Criteria; BIC: Bayesian Information Criterion; aBIC: sample-size adjusted BIC; LMR: lo-mendell-rubin; BLRT: Bootstrapped-likelihood ratio test; 1C: one-class; 2C: two-class; 3C: three-class; 4C: four-class; 5C: five-class.

**Fig 1:**
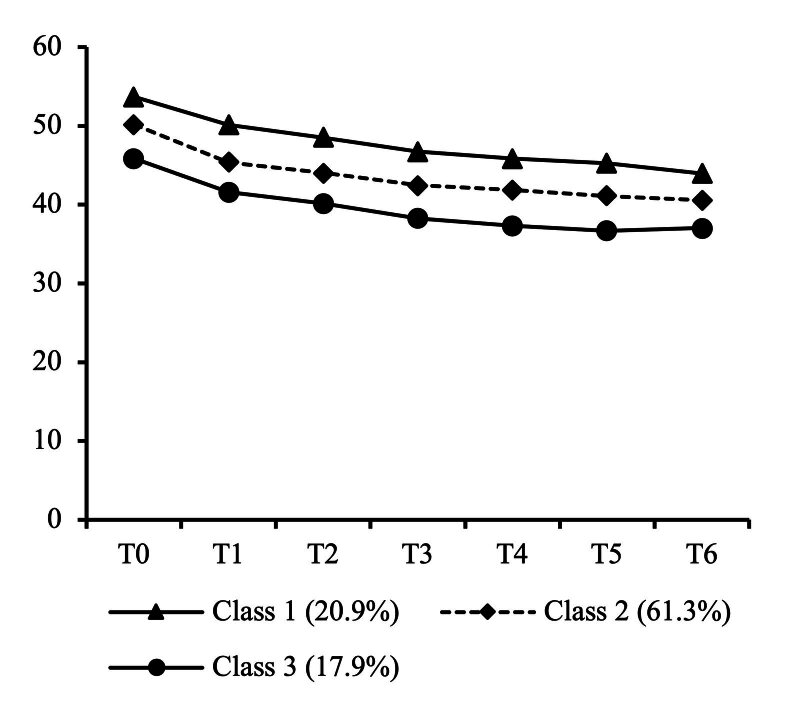
Trajectory categories of PNI in HNC radiotherapy patients.

The PNI trajectory categories resulting from the LCGM model fit are shown in Fig 1. Class 1 was named “PNI high-level group” (the highest initial value with a slowly decreasing trajectory), with 98 patients accounting for 20.9%; Class 2 was termed “PNI medium-level group” (initial value higher than the critical value with a slowly decreasing trajectory), with 288 patients accounting for 61.3%; and Class 3 was named “PNI low-level group” (initial value close to the critical value with a slowly decreasing trajectory), with 84 patients accounting for 17.9%.

### Baseline Characteristics of PNI Trajectories in HNC Radiotherapy Patients 

Table 2 shows the descriptive statistics of the covariates that may impact the classification results. The results showed statistically significant differences in age, BMI, educational level, occupational status, years of smoking, nasogastric intubation, erythrocytes, hemoglobin, lymphocytes, neutrophil percentage, total protein, and albumin.

### Baseline Characteristics of Severe RIOM in HNC Radiotherapy Patients

Severe RIOM occurred in 21.3% of patients during radiotherapy. The corresponding proportions of severe RIOM in each trajectory category were: “PNI high-level group”, 12.0%; “PNI medium-level group”, 62.0%; “PNI low-level group”, 26.0%. As shown in Table 3, variables affecting the occurrence of severe RIOM include PNI trajectory categories, occupational status, years of betel nut chewing, cancer type, disease duration (years), degree of differentiation, number of comorbidities, diabetes, surgery, nasogastric intubation, radiotherapy dose, platelets, monocytes, total protein, albumin, and globulin.

**Table 3:** Baseline characteristics of severe RIOM

Variables	All (n=470)	Severe RIOM	p
No	Yes
PNI trajectory categories				0.009
PNI high-level group	98 (20.9%)	86 (23.2%)	12 (12.0%)	
PNI medium-level group	288 (61.3%)	226 (61.1%)	62 (62.0%)	
PNI low-level group	84 (17.9%)	58 (15.7%)	26 (26.0%)	
Gender				0.112
Male	382 (81.3%)	295 (79.7%)	87 (87.0%)	
Female	88 (18.7%)	75 (20.3%)	13 (13.0%)	
Age in years				0.093
≤40	89 (18.9%)	75 (20.3%)	14 (14.0%)	
41~65	330 (70.2%)	260 (70.3%)	70 (70.0%)	
≥66	51 (10.9%)	35 (9.5%)	16 (16.0%)	
BMI				0.195
<18.5	25 (5.3%)	17 (4.6%)	8 (8.0%)	
18.5~23.9	264 (56.2%)	205 (55.4%)	59 (59.0%)	
24.0~27.9	136 (28.9%)	108 (29.2%)	28 (28.0%)	
≥28.0	45 (9.6%)	40 (10.8%)	5 (5.0%)	
Education level				0.388
Primary school and below	84 (17.9%)	65 (17.6%)	19 (19.0%)	
Junior high school	156 (33.2%)	117 (31.6%)	39 (39.0%)	
High school or junior college	142 (30.2%)	118 (31.9%)	24 (24.0%)	
College and above	88 (18.7%)	70 (18.9%)	18 (18.0%)	
Marital status				1.000
Married	445 (94.7%)	350 (94.6%)	95 (95.0%)	
Unmarried	21 (4.5%)	17 (4.6%)	4 (4.0%)	
Divorced or widowed	4 (0.9%)	3 (0.8%)	1 (1.0%)	
Occupational status				0.000
Full-time job	217 (46.2%)	167 (45.1%)	50 (50.0%)	
Unemployed	31 (6.6%)	21 (5.7%)	10 (10.0%)	
Retirement	51 (10.9%)	40 (10.8%)	11 (11.0%)	
Farmer	137 (29.1%)	123 (33.2%)	14 (14.0%)	
Other	34 (7.2%)	19 (5.1%)	15 (15.0%)	
Years of smoking				0.500
0	204 (43.4%)	165 (44.6%)	39 (39.0%)	
1~10	58 (12.3%)	43 (11.6%)	15 (15.0%)	
≥11	208 (44.3%)	162 (43.8%)	46 (46.0%)	
Years of drinking				0.098
0	313 (66.6%)	255 (68.9%)	58 (58.0%)	
1~10	54 (11.5%)	38 (10.3%)	16 (16.0%)	
≥11	103 (21.9%)	77 (20.8%)	26 (26.0%)	
Years of betel nut chewing				0.000
0	386 (82.1%)	325 (87.8%)	61 (61.0%)	
1~10	58 (12.3%)	30 (8.1%)	28 (28.0%)	
≥11	26 (5.5%)	15 (4.1%)	11 (11.0%)	
Place of residence				0.820
Urban	201 (42.8%)	157 (42.4%)	44 (44.0%)	
Rural	269 (57.2%)	213 (57.6%)	56 (56.0%)	
Cancer type				0.000
Oral cancer	121 (25.7%)	61 (16.5%)	60 (60.0%)	
Nasopharyngeal cancer	295 (62.8%)	273 (73.8%)	22 (22.0%)	
Laryngeal cancer	21 (4.5%)	18 (4.9%)	3 (3.0%)	
Other	33 (7.0%)	18 (4.9%)	15 (15.0%)	
Disease duration (years)				0.008
<1	437 (93.0%)	351 (94.9%)	86 (86.0%)	
1~3	23 (4.9%)	13 (3.5%)	10 (10.0%)	
≥4	10 (2.1%)	6 (1.6%)	4 (4.0%)	
Degree of differentiation				0.000
Well differentiated	69 (14.7%)	36 (9.7%)	33 (33.0%)	
Moderately differentiated	80 (17.0%)	43 (11.6%)	37 (37.0%)	
Poorly differentiated	91 (19.4%)	77 (20.8%)	14 (14.0%)	
Undifferentiated	230 (48.9%)	214 (57.8%)	16 (16.0%)	
Cancer stage				0.696
I	6 (1.3%)	5 (1.4%)	1 (1.0%)	
II	34 (7.2%)	25 (6.8%)	9 (9.0%)	
III	193 (44.1%)	149 (40.3%)	44 (44.0%)	
IV	237 (50.4%)	191 (51.6%)	46 (46.0%)	
Number of comorbidities				0.048
0	61 (13.0%)	50 (13.5%)	11 (11.0%)	
1~4	268 (57.0%)	216 (58.4%)	52 (52.0%)	
5~10	130 (27.7%)	99 (26.8%)	31 (31.0%)	
≥11	11 (2.3%)	5 (1.4%)	6 (6.0%)	
Diabetes				0.002
Yes	66 (14.0%)	42 (11.4%)	24 (24.0%)	
No	404 (86.0%)	328 (88.6%)	76 (76.0%)	
Surgery				0.000
Yes	180 (38.3%)	105 (28.4%)	75 (75.0%)	
No	290 (61.7%)	265 (71.6%)	25 (25.0%)	
Simultaneous chemoradiotherapy			0.129
Yes	296 (63.0%)	240 (64.9%)	56 (56.0%)	
No	174 (37.0%)	130 (35.1%)	44 (44.0%)	
Nasogastric intubation				0.000
Yes	38 (8.1%)	16 (4.3%)	22 (22.0%)	
No	432 (91.9%)	354 (95.7%)	78 (78.0%)	
Radiotherapy dose	69.96 (66.00, 69.96)	69.96 (69.96, 69.96)	66.00 (64.00, 69.96)	0.000
Laboratory test results				
Leukocytes	5.10 (4.10, 6.40)	5.20 (4.18, 6.43)	4.80 (4.00, 6.30)	0.218
Erythrocytes	4.13 (3.75, 4.58)	4.11 (3.72, 4.58)	4.19 (3.88, 4.58)	0.230
Hemoglobin	125.48±16.79	124.98±17.41	127.32±14.19	0.166
Platelets	240.00 (188.00, 296.25)	246.00 (193.00, 307.50)	210.00 (176.50, 254.00)	0.000
Neutrophils	3.00 (2.30, 4.00)	3.00 (2.20, 4.03)	3.05 (2.40, 3.80)	0.851
Lymphocytes	1.40 (1.10, 1.70)	1.40 (1.10, 1.70)	1.30 (1.00, 1.70)	0.218
Monocytes	0.50 (0.40, 0.60)	0.50 (0.40, 0.60)	0.40 (0.30, 0.58)	0.000
Neutrophil percentage	59.56±10.82	59.23±10.90	60.78±10.50	0.204
Total protein	72.00 (68.55, 75.23)	72.60 (69.20, 75.53)	70.25 (66.03, 73.45)	0.000
Albumin	43.30 (41.20, 45.40)	43.40 (41.60, 45.73)	41.85 (39.45, 44.48)	0.000
Globulin	28.55 (25.90, 31.40)	28.70 (26.40, 31.50)	26.75 (24.25, 30.73)	0.003
Creatinine	72.00 (62.30, 82.00)	73.00 (63.00, 82.85)	69.80 (59.25, 79.75)	0.114


### Logistic Regression Model Relating PNI Trajectory Categories to Severe RIOM

Table 4 shows the logistic regression models relating PNI trajectories to severe RIOM. The risk of severe RIOM was statistically significantly higher in the “PNI medium-level group” and “PNI low-level group” with OR 2.174 (95% CI 0.98-4.822) and OR 3.45 (95% CI 1.212-9.815), respectively. However, the association between the “PNI medium-level group” and severe RIOM was attenuated and lost statistical significance (p>0.05). “PNI low-level group” and oral cancer remained statistically significant and were risk factors for developing severe RIOM.

**Table 4:** Logistic regression model relating PNI trajectory categories to severe RIOM

Variables	β	Standard error	OR	95%CI	Wald x^2^	p
PNI trajectory categories				
PNI high-level group	-	-	-	-	-	-
PNI medium-level group	0.777	0.406	2.174	0.980-4.822	3.651	0.056
PNI low-level group	1.238	0.534	3.450	1.212-9.815	5.387	0.020
Years of betel nut chewing				
0	-	-	-	-	-	-
1~10	0.136	0.406	1.146	0.517-2.540	0.113	0.737
≥11	-0.177	0.512	0.838	0.307-2.284	0.119	0.730
Occupational status			
Full-time job	-	-	-	-	-	-
Unemployed	0.342	0.527	1.408	0.501-3.953	0.422	0.516
Retirement	-0.293	0.481	0.746	0.291-1.915	0.371	0.543
Farmer	-0.552	0.391	0.576	0.268-1.238	1.997	0.158
Other	0.229	0.473	1.257	0.498-3.173	0.234	0.629
Cancer type						
Oral cancer	-	-	-	-	-	-
Nasopharyngeal cancer	-0.931	0.730	0.394	0.094-1.650	1.624	0.203
Laryngeal cancer	-2.296	0.739	0.101	0.024-0.428	9.658	0.002
Other	-0.144	0.509	0.866	0.320-2.346	0.080	0.777
Disease duration (years)						
<1	-	-	-	-	-	-
1~3	0.460	0.553	1.585	0.537-4.680	0.694	0.405
≥4	-0.395	0.757	0.673	0.153-2.969	0.273	0.601
Degree of differentiation						
Well differentiated	-	-	-	-	-	-
Moderately differentiated	0.149	0.396	1.161	0.534-2.523	0.142	0.706
Poorly differentiated	-0.770	0.549	0.463	0.158-1.358	1.968	0.161
Undifferentiated	-0.818	0.724	0.441	0.107-1.824	1.276	0.259
Number of comorbidities						
0	-	-	-	-	-	-
1~4	0.143	0.450	1.153	0.478-2.785	0.100	0.751
5~10	0.651	0.509	1.918	0.708-5.197	1.638	0.201
≥11	0.952	0.890	2.592	0.452-14.843	1.144	0.285
Diabetes						
No	-	-	-	-	-	-
Yes	0.206	0.365	1.228	0.601-2.509	0.318	0.573
Surgery						
No	-	-	-	-	-	-
Yes	0.630	0.451	1.878	0.777-4.542	1.957	0.162
Nasogastric intubation						
No	-	-	-	-	-	-
Yes	0.300	0.449	1.350	0.560-3.252	0.447	0.504
Radiotherapy dose	-0.001	0.026	0.999	0.949-1.052	0.001	0.972
Laboratory test results						
Platelets	-0.002	0.002	0.998	0.994-1.002	0.799	0.371
Monocytes	0.175	0.227	1.191	0.763-1.860	0.593	0.441
Total protein	0.635	1.019	1.888	0.256-13.901	0.389	0.533
Albumin	-0.643	1.019	0.525	0.071-3.875	0.398	0.528
Globulin	-0.724	1.020	0.485	0.066-3.578	0.504	0.478


## Discussion

In this study, more than half of the patients (61.3%) were in the “PNI medium-level group”. Of note, there were almost equal proportions of the “PNI high-level group” and “PNI low-level group” at 20.9% and 17.9%, respectively. Compared with the “PNI high-level group”, the patients in the “PNI medium-level group” and “PNI low-level group” had a higher risk of severe RIOM. Furthermore, we determined that PNI trajectory categories and cancer type were independent risk factors for severe RIOM.

Currently, the nutritional status of patients with malignant tumors is a focus of clinical attention, and the nutritional problems of HNC patients undergoing radiotherapy are particularly prominent due to tumor location and the hypermetabolic state, as well as adverse reactions caused by the treatment.^26^ Malnutrition is a common problem in HNC patients during radiotherapy. Critical weight loss due to malnutrition may lead to shifting of radiotherapy targets, changes in the radiotherapy plan, prolongation of treatment cycles, and increased healthcare costs, as well as reduced tolerance of treatment, decreased immune function and life quality, increased risk of infections and mortality in patients.^18,22,23,24^ In addition, the radiation-induced toxicities of mucosa, salivary glands, pharynx, and esophagus in HNC patients with malnutrition are often worse.^6^ In other words, malnourished patients are at greater risk of developing severe RIOM, and patients who develop severe RIOM may further exacerbate malnourishment, with the two interacting to lead to a poor prognosis. Regrettably, there is no consensus on the criteria for assessing malnutrition.

The results of the available studies prove a correlation between oral mucositis induced by radiotherapy for HNC and nutritional status.^30,31^ PNI is calculated based on the lymphocyte counts and albumin level, which is simple and objective and has been widely used in clinical practice. PNI is an index that reflects chronic inflammation, immune system, and nutritional status.^11^ On the one hand, tumor-infiltrating lymphocytes are essential to cellular immunity and can eliminate tumor cells through cellular immunity.^25^ On the other hand, total lymphocyte counts in the peripheral blood in tumor patients often decrease after stressful events such as surgery, trauma, radiotherapy, etc. If it continues to decline, it may promote the secretion of glucocorticoids and adrenaline, which causes the redistribution of lymphocytes in lymphoid tissues, accelerates apoptosis, and produces a deleterious inflammatory response.^13^ The liver mainly secretes albumin, and its serum level effectively reflects the protein content, liver function reserve, and nutritional status. As an ordinary biomarker of trophic status, low albumin levels are associated with chronic inflammation that triggers IL-1, TNF-α, and other cytokine stimulation.^7^ This study proves that PNI can be used as a biomarker to predict the development of severe RIOM. This suggests that aggressive treatment aimed at controlling lymphocyte counts and albumin levels may ameliorate the PNI level more favourably and reduce the severity of oral mucositis.

In addition, this study found that laryngeal cancer had a lower risk of severe RIOM than patients with oral cancer. Due to the different primary sites of tumors, radiotherapy target areas are different. The closer the tumor target area is to the oral cavity and pharynx, the higher the irradiation dose to the corresponding mucosa and the more severe the occurrence of mucositis.^34^

This study has some limitations. First, all patients were followed-up from before radiotherapy to the end of radiotherapy. However, most patients continued suffering from oral mucositis after the completion of radiotherapy. Future studies should extend the follow-up period. Second, this was a retrospective longitudinal study, and the data recording may have been subject to information bias. Finally, the present study is a single-center study, and generalization of the findings may be limited. Multicenter and prospective studies should be conducted to explore the correlation between PNI trajectory categories and severe RIOM in the future.

## Conclusion

This study examined PNI trajectory categories in HNC patients undergoing radiotherapy. Three trajectory categories were identified, “PNI high-level group”, “PNI medium-level group” and “PNI low-level group”, in which patients’ PNI values declined over radiotherapy duration. In conclusion, our study demonstrated that PNI may be an independent, significant predictor of severe RIOM. Patients with head and neck cancer who have a lower PNI are at higher risk of severe RIOM.

## ACKNOWLEDGMENT

We thank Ward 65, Department of Head and Neck Cancer Radiotherapy, Xiangya Hospital Central South University for supporting this study. Funding for this project was provided by the Hunan Provincial Health Commission (number: D202309038051).
